# Partial Paralysis from Reflex Irritation

**Published:** 1871-01

**Authors:** 


Partial Paralysis from Reflex Irritation.—Dr L. A. Sayre read a paper at the last meeting of the American Association, giving his observation on the production of partial paralysis of certain muscles of the lower limbs by irritation of the glans penis from congenital phymosis.

He abbreviates the history of six cases in which he found this condition to exist.

The patients were all afflicted with a variety of nervous symptoms beside the paralysis, and all, on circumcision, were relieved. He says he is satisfied "that many of the cases of irritable children, with restless sleep and bad digestion, which is often attributed to worms, is solely due to the irritation of the nervous system, caused by an adherent or constricted prepuce."

The first case he had was a boy of five years, who had such paralysis of the quadriceps that the legs were flexed at an angle of 45 degrees. The doctor, in applying electricity to the paralyzed muscles, discovered the irritation of the penis. This organ was very sore, and the slightest irritation, as of the clothing, or even a jarring of the body, caused an erection. Circumcision was performed, and the child began immediately to improve in general health, slept well at night, and improved in appetite. In five weeks he could walk alone, with his limbs quite straight.

The second case was of a lad of 14, whom he had treated for months for slight paralysis of his legs, when it was found he had phymosis and constant irritation of the glans. He had nocturnal emissions and erection on the most trivial irritation. He was circumcised, and in six weeks had entirely recovered.

The third case was of a boy of 15, who had nervousness and "fainting fits;" he had "falling fits" because "his legs would not hold him up." He had every night painful erection and very frequent emissions; the prepuce was enormously redundant, and all the parts excessively irritable. In six weeks after circumcision, he was nearly well.

The other three cases had hip disease, which was found to have been lighted up by repeated falls due to the partially paralyzed condition of the legs from irritation of the penis. The patients were excessively nervous. On circumcision, they were relieved of all the symptoms, save those caused by the lesion of the hip.—*Transactions of the Association.*

